# “Betting on nature” or “betting on others”: anti-coordination induces uniquely high levels of entropy

**DOI:** 10.1038/s41598-018-21962-1

**Published:** 2018-02-23

**Authors:** Gabriele Chierchia, Rosemarie Nagel, Giorgio Coricelli

**Affiliations:** 1 Max Planck Institute for Human Cognitive and Brain Science, department of Social Neuroscience, Leipzig, Germany; 2 University of Southern California, Department of Economics, Los Angeles, USA; 3 University of Trento, Center for Mind/Brain Sciences, Rovereto, Italy; 4 ICREA, Universitat Pompeu Fabra, Department of Economics, Barcelona, Spain

## Abstract

Uncertainty in the form of risk or ambiguity can arise from the interaction with nature and other players, while strategic uncertainty arises only in interactions with others. Here, we systematically compare binary decisions between a safe option and a potentially higher paying but uncertain option in four experimental conditions with the same potential monetary outcomes: coordination vs. anti coordination games, as well as risky and ambiguous lotteries. In each condition, we progressively increase the value of the safe option and measure subjects’ certainty equivalents (i.e., the specific safe payoff-threshold that makes a subject indifferent between the two options). We find that anti-coordination games and ambiguous lotteries elicit equally high aversion to uncertainty, relative to the other domains. In spite of this similarity, we find that subjects alternate between the safe and uncertain options much more frequently, thus displaying higher entropy, under anti-coordination relative to any of the other environments. These differences are predicted by theories of recursive reasoning in strategic games (e.g., thinking what others think we think etc.). Indeed, this can occur when interacting with intentional counterparts, but not with nature.

## Introduction

“Half a century ago, when decision theory and game theory were young, it was common to perceive a dichotomy between (i) games against nature, in which the “adversary” is a neutral “nature”—and (ii) strategic games, in which the adversary is an interested party […]. No need was seen to reconcile or even relate the approaches. In the ensuing years, the dichotomy gradually disappeared. It was recognized that games against nature and strategic games are in principle quite similar, and can—perhaps should—be treated similarly. Specifically, a player in a strategic game should be able to form subjective probabilities over the strategies of the other players, and then choose his own strategy so as to maximize his expected utility with respect to these subjective probabilities.” (Aumann and Dreze^1^).

Many financial and daily decisions involve “betting” on others. Deciding whether to bet on what may turn out to be a speculative bubble, to join a rebellion, to invest in a new technology, critically depends on what we think others will do: if others bet on the same bubble it may hold long enough to provide revenue, if others also join the rebellion it may succeed, and if they invest in the same technology it might become the new standard^2^. In the opening passage above, Aumann and Dreze ask the following question: can and should such social decisions be treated similarly to bets on “blind nature”? On the one hand they could: agents would maximize their utility based on their beliefs on which states of the worlds will occur, whether these depend on others or not. On the other hand, in games against nature, this state of the world is the result of a mechanistic process, while in games against others, it is the result of a motivated decision process, where others may form beliefs about what we will choose, or iteratively, about what we think they will choose etc. With this in mind, Aumann and Dreze argue, the structure of the game can change. Hence the question: does betting on others fundamentally change the way humans face uncertainty?

Aumann and Dreze illustrate this problem formally. Here we investigate the matter empirically, by systematically comparing “how” experimental participants make decisions (and not just “which” decisions they make) in various environments involving either another player or nature (i.e., “lotteries”), while keeping potential monetary outcomes identical across environments. Among the many types of “games against others”, we focus on coordination games because “risk” has already been invoked to explain the way agents may face these problems^3^. In fact, while a subset of interactions with others can be “solved” by deductive reasoning, that is, by Nash-equilibrium (“NE”) analysis, coordination games involve multiple NE, thus raising a problem of “equilibrium *selection*”^4^, or what has also been called “strategic uncertainty”^5^. We asked whether the strategic uncertainty encountered in a *coordination game* is similar to that encountered under the more traditional notion of risk and uncertainty, one that conceptualizes (and measures) uncertainty through the use of lotteries^6^.

Among coordination games, we further focus on the “stag hunt” game (henceforth “SH”, also called assurance game) and the entry game (“EG”, also called the chicken game), because these are paradigmatic of two “opposite declinations” of coordination. SHs have in fact been called the “building block” of situations with “strategic complementarities” (Camerer^2^): games in which agents have an incentive to *match* their choices, and which are known to foster cooperation^7,8^. For instance, in the rebellion case sketched above, either all rebel or no one does, but mismatched choices are costly. EGs on the other hand involve “strategic substitutability”: environments in which agents have an incentive to mismatch their choices, and which can be conducive to competition^9,10^. For instance, all might prefer to be the few ones on the freeway, though doing so all at the same time could lead to a traffic jam. Similarly, in many markets, agents know that if too many others invest in the same asset (i.e., “enter” the market), there will be a disadvantageous price war^2^. In synthesis, SHs involve pure coordination - situations in which agents should match their actions - while EGs involve anti-coordination - situations in which agents should choose opposite actions.

In our stag hunt variant - adapted from Heinemann *et al*.^11^ (see Camerer^2^, and Devetag and Ortmann^12^, for reviews on other variants) - pairs of anonymous players choose between one of two options, without knowing what the other chooses, without communication and without being informed about previous outcomes (to preclude learning by experience). One of these two options is generally “low paying” but “safe” and if a participant chooses this option, he/she obtains a given Euro amount for sure, for instance €2.00, regardless of what the other chooses. We thus call this option the “SP” (as in “safe” or “sure” payoff). On the other hand, one can obtain a relatively higher gain, for instance €15.00, by selecting the other option. However, this higher payoff is obtained *only if* one’s counterpart also chooses this “uncertain option” (henceforth, “UP”), that is, if one chooses the UP alone, he/she earns nothing at all. As mentioned above, deductive rationality (i.e., game theory) has little to say as to what to choose in SHs – though see Carlsson and Damme^13^ and Morris and Shin^14^ for notable game theoretic refinements introducing uncertainty and signals about payoffs which can lead to a unique equilibrium in such games -, if not that agents should try to “match” their choices: either “both choose the SP”, or “both choose the UP”, but mismatched choices are disadvantageous. Indeed, these are the two (pure-strategy) NEs of the game.

However, following Harsanyi & Selten’s notion of “risk-dominance”^3^, there is another strong intuition that, even retaining the SH structure, specific payoff differences may matter very much. Indeed, as we illustrate below, empirically, they do^11,15,16^. The example provided above of a SH with SP = €2.00 (for sure) and UP = €15.00 (if both choose it, €0, otherwise) should seem trivial to most and, indeed, most experimental participants coordinate successfully on the (Pareto-efficient) UP option in a SH with those parameters^11^. However, what if (all else being equal) the value of the SP was raised to €8.00? Empirically, many participants cease to choose the UP and now choose the SP. It follows that each participant will have his or her idiosyncratic “threshold” and that this can be measured by having participants make repeated choices (without feedback) over a sensible range of parameters (i.e., increasing SP values), and observing at which point they switch between options (much as in the tradition of establishing certainty equivalents or risk premiums, as well perceptual thresholds in classic psychophysics experiments). In line with this, Heinemann and colleagues^11^ compared choices in a SH with choices in a lottery with identical payoffs (that is, instead of “betting on others” participants were to bet on a lottery, which “selects” UP or SP with a given probability) and showed that participants clearly used threshold strategies across both domains.

Returning to EGs, these involve strategic anti-coordination, but can nonetheless be represented in a superficially very similar way to SHs. In fact, to transform the SH above into an EG, as shown by Nagel *et al*.^17^, it is sufficient to only alter the consequences of choosing the UP option, all else being equal: in EGs participants earn the higher payoff only if they choose the UP and their counterpart “does not”. Like SHs, EGs have two pure strategy NE (either “I choose UP and you don’t” or vice versa) and one mixed-strategy equilibrium. However, based on cognitive hierarchy models of reasoning^18–20^, we hypothesize that subjects should display a particular decision pattern in EGs. Specifically, we expect depth of reasoning to change choices, and thus increase entropy, in EGs, but not SHs, as we illustrate below.

Cognitive hierarchy models assume that subjects may perform different levels of strategic/recursive reasoning in interdependent decision problems: “0-level” players perform no strategic reasoning at all, and choose each available option with uniform probability (i.e., as a random lottery). Level-1 players assume that they are interacting with level-0 players, level-2 players assume they are mostly playing level-1 players, but also with some level 0 players, and so forth. Let us now return to our previously exemplified SH parameters (SP = €2.00 and UP = €15.00), and consider what cognitive hierarchy models would call a “level-1 thinker”. Such a level-1 thinker assumes that his or her counterpart will be a “level-0 thinker”, that is, a player who chooses randomly, by selecting SP or UP with a uniform distribution (i.e., in our binary choice case, p = 0.5). Since choosing the UP when playing with a randomizing counterpart yields €15.00 or 0 with equal probability, the expected value of choosing UP for a risk neutral level-1 thinker is €7.50; since this is higher than the alternative SP of €2.00, a level-1 thinker is predicted to choose the UP in a SH with these parameters. Lets now consider a level-2 thinker in the exact same game. This player now assumes that his counterpart could be a level-1 player and thus that such a counterpart will choose the UP (given the reasoning above). This in turn provides assurance to the level-2 player, who now has an incentive to choose the UP as well (in order to match the choice of the level-1 counterpart). Importantly, increasing levels of reasoning further doesn’t alter this pattern because level-2 players will want to match level-1 players, and level-3 players will match level-2 players etc^20^. In short, increasing levels of reasoning doesn’t alter UP/SP choices in SHs: players only need to think of the probability with which the counterpart choose the UP choice.

However, this is not the case in EGs. In fact, if one performs the same reasoning above on an EG with identical payoffs it becomes clear that, as in a SH, a level-1 player is predicted to choose UP but that, in contrast to SHs, a level 2 player will now “switch” his/her choice by choosing SP. In fact, if a level-2 player expects his/her counterpart to choose the UP in an EG with SP = €2.00, this no longer provides assurance like in the SH, rather, it provides deterrence from attempting to choose the UP, since both players would earn nothing in this case. Moreover, agents would be expected to mentally alternate back and forth between the SP and UP choices as their depth of reasoning increases. We thus reasoned that, since hierarchical reasoning cannot be applied to “blind nature” (which, in a way, is always level-0) and does not alter choices in SHs, then subjects should change their choices more frequently in EGs, relative to any of the other environments. Operationally, we thus predicted that not only subjects may be likely to have distinct beliefs about the choices of their counterparts in SHs and EGs (and thus display distinct thresholds), but also that, especially in EGs, subjects would more frequently switch between SP and UP choices. In other words, especially under anti-coordination, we expected to observe more frequent violations of monotonicity, or entropy. Furthermore, to investigate whether this could be simply due to EGs fostering more random behavior or noise we first investigated whether anti-coordination would be associated with increased decision times - which are considered a typical hallmark of deliberative cognitive processes, cognitive load^21,22^ or, more simply, task difficulty^23^. Second, we assessed how switch frequency affected expected payoff in EGs, relative to the other decision environments. Third, we evaluated whether the specific switch frequencies we observe are consistent with known distributions of levels or reasoning in games^20^.

Finally, irrespective of levels of reasoning, subjects might simply expect more variable behavior from their counterparts in EGs than SHs, which might decrease their confidence in their own judgments (i.e., their beliefs). In non-social decision environments, decreased confidence has frequently been labeled “ambiguity”, and it has been associated with aversion to uncertainty. For instance, as originally suggested by Ellsberg^24^, subjects may prefer to bet on an urn that contains precisely 50 “winning balls” and 50 losing ones, rather than an urn containing an unspecified mixture of the two colors. Since in the second but not the first urn subjects may speculate over what the success probability is, ambiguity has also been operationalized as variance in second-order probabilities^25^. In line with this, previous studies have documented aversion to uncertainty in anti-coordination games or affinity for uncertainty in coordination games^26–28^, suggesting there may be a link between anti-coordination and ambiguity^26^. However those studies only focused on betting preferences and not on entropy. We thus aimed to systematically compare both measures in coordination vs. anti-coordination games, as well as risky vs. ambiguous lotteries. This ambiguity also parallels the missing objective success probability in the coordination games, and as a contrast to it, we further introduced risky lotteries, in which the success probability was known.

**Figure 1:**
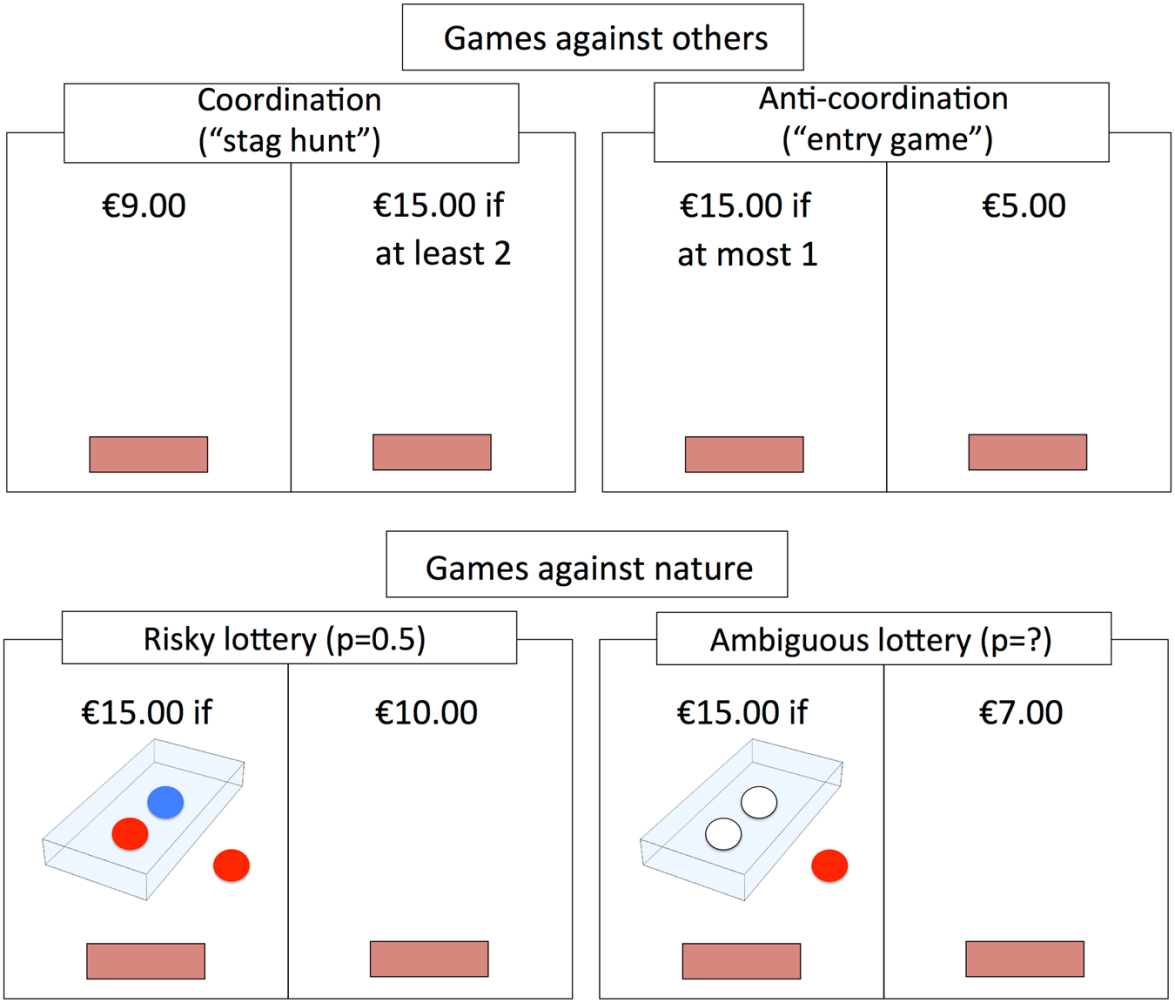
Experimental design. Sample screens for each of the four decision environments in which participants chose between a low paying but safe option (a safe option, e.g., “SP”) and an option worth more or nothing (thus an uncertain option, “UP”), depending on a risky or uncertain outcome. The UP was always worth €15.00 or €0, while the value of the SP varied from trial to trial (covering the whole range between 0 and €15.00 equally for each environment). *Top row:* “games against others” involved one of two coordination games, in which the outcome of the uncertain option depended on the choice of another player. *Left:* a coordination game (a “stag hunt”), in which both participants could obtain the highest payoff by both choosing the UP option. However, if one chose the UP alone, he/she earned nothing. *Right:* an anti-coordination game (an “entry game”), in which participants could only obtain the high payoff if they chose the UP option but their counterpart did not. If both chose the UP, both earned nothing at all. *Bottom row:* “games against nature” involved one of two lottery extractions, in which the outcome of the risky or uncertain option depended on an extraction from a real urn. *Left:* in a risky lottery condition, the probability of winning was known (and occurred with a probability of 0.5). *Right:* in an ambiguous lottery condition, the probability of winning was unknown.

We experimentally investigate this by analyzing incentivized choices under uncertainty involving either real lotteries (i.e., risk or ambiguity) or others (i.e., coordination vs. anti-coordination) (Fig. 1, see Methods for details). In each environment, subjects were to choose between a safe option, worth a given monetary amount (e.g., €7.00), and an uncertain option (always worth €15.00 or €0). As proxies for uncertainty, we compared the probability of choosing the uncertain option (as opposed to the safe one) in each environment and then measured subjects’ “revealed uncertainty” by gradually increasing the value of the safe option (without feedback) and computing the value at which they “switched” between the two options, i.e., their “certainty equivalents”. Importantly, we also measure subjects’ “entropy”, as well as their decision times (see Methods for details).

## Results

**Figure 2:**
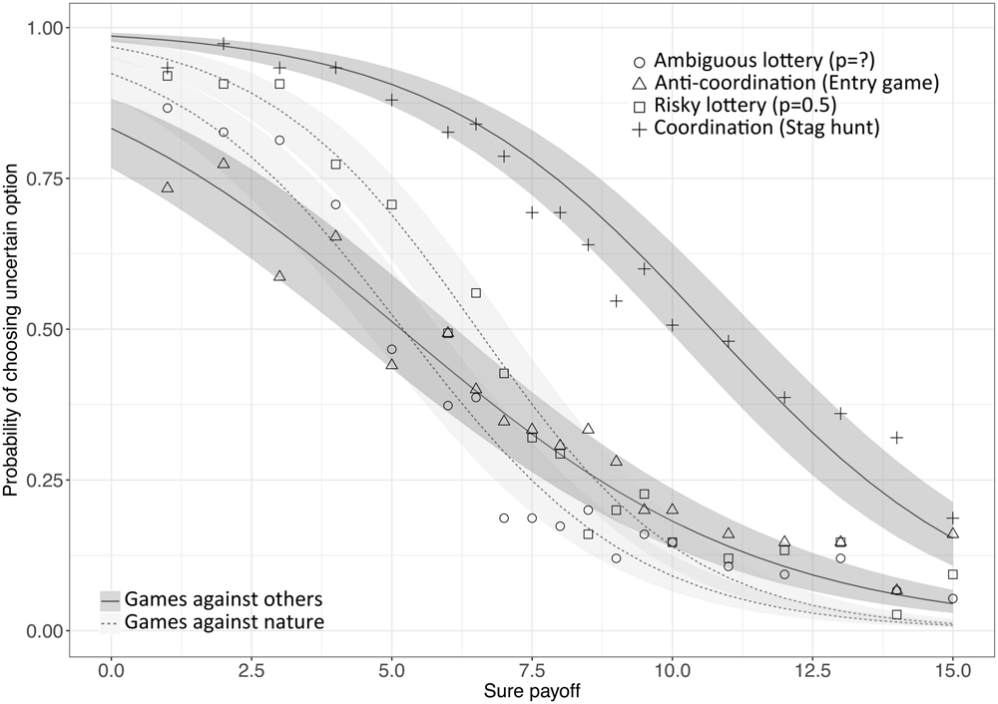
Estimated probability of choosing an uncertain option (“UP”) (y-axis) relative to a lower paying but safer alternative (“SP”) (x-axis) in games against others (darker shades) or games against nature (lighter shades). Symbols represent the raw percentages of UP choices observed in each of the four decision environments, for each SP value: coordination games (i.e., stag hunts, crosses), anti-coordination games (i.e., entry games, triangles), lotteries with risky/known success probability (of p = 0.5, squares), or ambiguous/unknown success probability (circles). Shaded ribbons represent 95% confidence intervals of the estimated fixed effects.

A generalized logistic hierarchical model revealed that in each decision environment increasing values of the SP significantly decrease the probability of choosing the UP option (i.e., all slopes are negative and significant, p_s_ < 0.001) (Fig. 2). However, the entry game significantly interacted with the SP term in predicting the likelihood of choosing the UP option (p < 0.001). Specifically, while at low SPs, the probability of choosing the UP was lower in entry games than either of the lotteries (p < 0.001), this pattern was reversed at high sure payoffs. The stag hunt also interacted more weakly, but significantly (p < 0.05) with the SP, suggesting that it especially increased the likelihood of choosing the UP at relatively higher SP values, as compared to the other decision environments. In contrast to this, the likelihood of choosing the UP did not differentially interact with increasing SPs in the two lotteries (i.e., their two slopes were similar), though the probability was overall significantly decreased by ambiguity.

**Figure 3:**
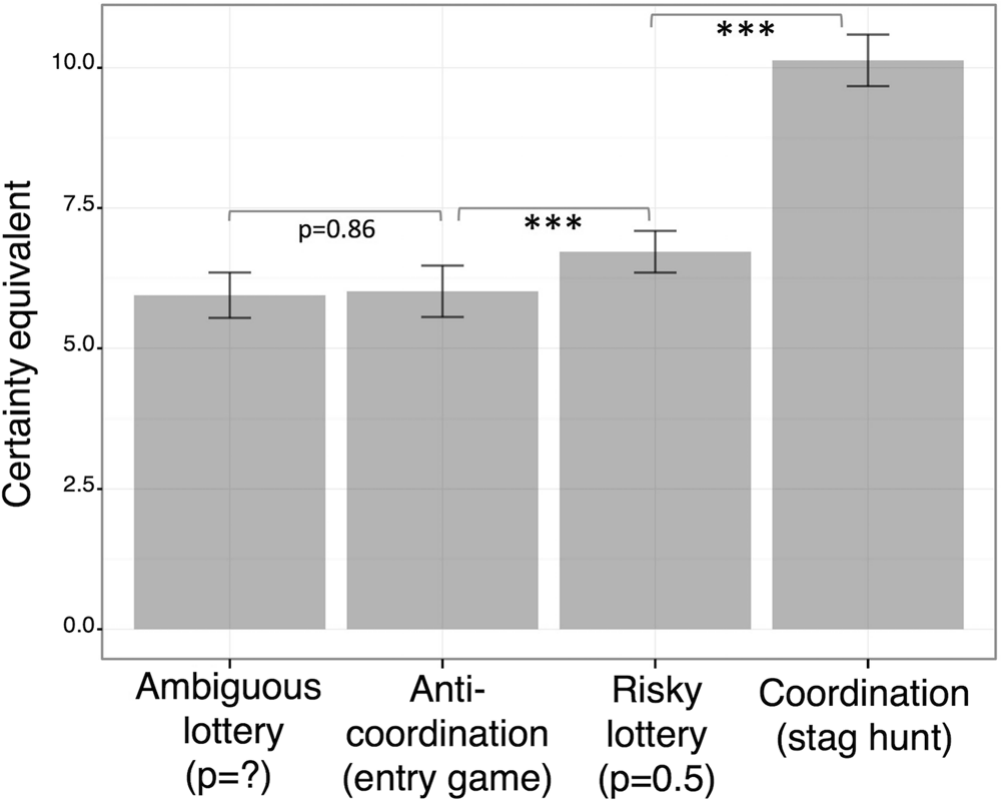
Average certainty equivalents across decision environments. For each participants and each decision environment, certainty equivalents were computed. Error bars represent standard error of the means. Asterisks represent significance levels of paired Wilcoxon signed-rank tests: ***p < 0.001.

In line with this, signed-rank tests confirmed that certainty equivalents (Fig. 3) were higher in the stag hunt than in any of the other environments (all p_s_ < 0.001) and were higher in the risky lotteries than then the ambiguous ones (p < 0.01). Interestingly however, certainty equivalents were non-dissociable between the entry game and the ambiguous lotteries (p = 0.86), thus yielding the following overall pattern of inequalities with regards to certainty equivalents: entry game = ambiguity <risky lotteries (p = 0.5) <stag hunt.

**Figure 4:**
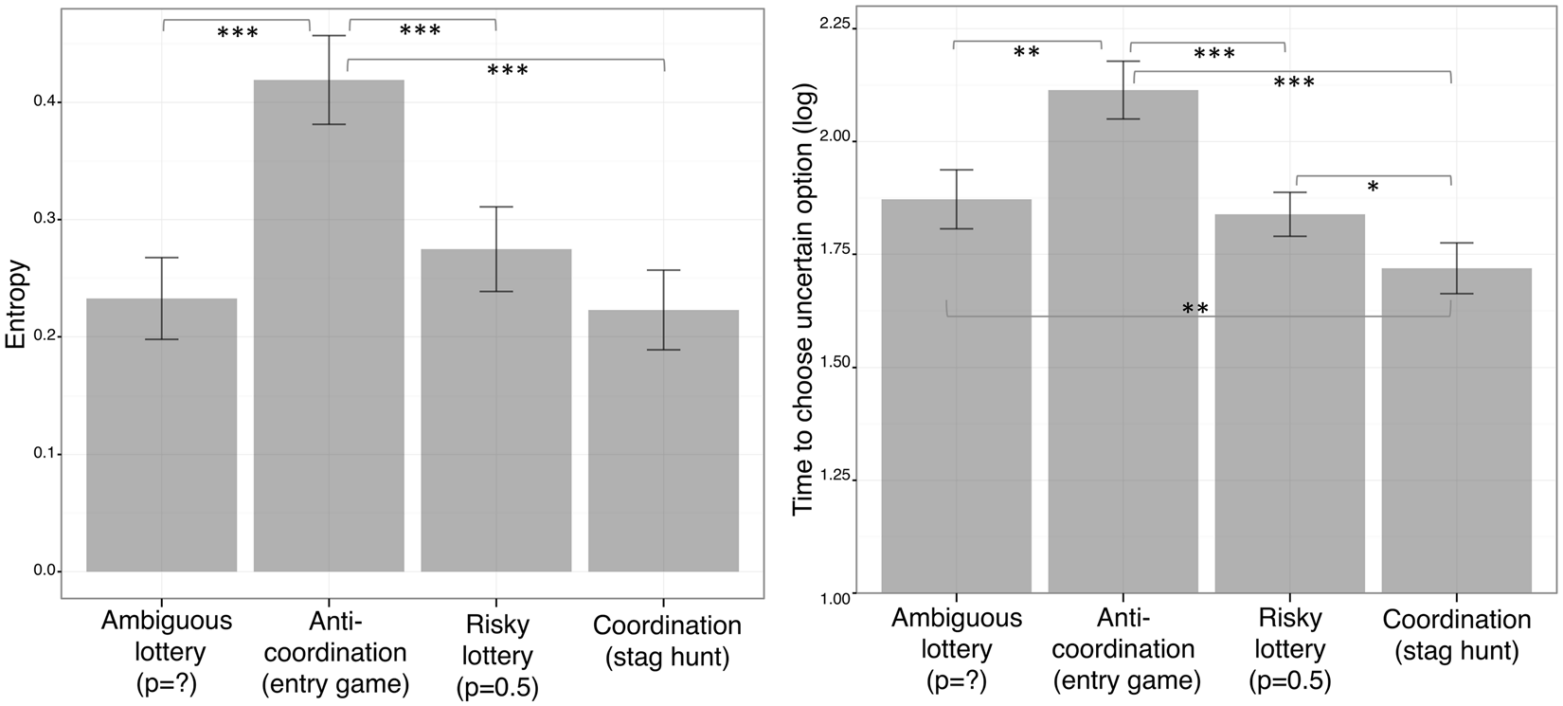
Left: entropy is increased in games involving anti-coordination (i.e., an entry game); relative to any of the other tested environments. Right: coordination and anti-coordination oppositely affect decision times, respectively facilitating and “disfacilitating” uncertain choices, relative to risky and ambiguous lotteries with the same potential monetary payoffs. Error bars represent standard error of the means. Asterisks represent significance levels of Wilcoxon signed-rank tests. ***p < 0.001, **p < 0.01, *p < 0.05.

All though the entry game and the ambiguous lotteries did not differ in terms of certainty equivalents, entropy was clearly higher in the entry game than any of the other environments (all p_s_ < 0.001). For instance, on average, participants switched choices 4 times in the entry game (~22%) and 2.7 times in the stag hunt (~14%) (the medians were 3 and 1 respectively), these different frequencies were highly significant (p <0.001). In contrast, entropy levels did not significantly differ between stag hunts, risky and ambiguous lotteries (all p_s_ > 0.25) (Fig. 4, left panel). Correspondingly, when participants chose the UP, they took more time to do so in the entry game than any of the other environments (all p_s_ < 0.01) (Fig. 4, right panel). Moreover, and conversely, UP choices were temporally facilitated in stag hunts, relative to any of the other environments (all p_s_ < 0.05).

We asked whether this observed average of 4 switches in the entry game is consistent with the average thresholds rates predicted by cognitive hierarchy theories for our game parameters. Following existing evidence regarding the distribution of levels of reasoning^20^, we assumed a Poisson distribution of k-level players with a lambda = 1.5. This distribution includes 22% level-0 players - which could be expected to randomly switch between SP and UP, thus on average 9 of 18 times – and 33% level-1 players – which should exhibit perfect threshold strategies (1 switch, at SP = 7.5, assuming risk-neutrality). Following the formulation proposed by Chong, Camerer and Ho^20^, it can than also be computed that, both level-2, which constitute 25% of players, and level-3 players (12%), should display 3 switches.

For instance, a risk-neutral level-2 players should switch from the UP to the SP at lower SP ranges than a risk-neutral level-1 player (specifically, at a value of SP = 3). Indeed, such a level-2 player believes that, for SP values between 4 and 7.5, there are too many level-0 and level-1 players choosing the UP, decreasing its expected value below the corresponding SP value. On the other hand, when level-2 players think level-1 players have now begun to choose the SP (i.e., at 7.5), level-2 players have an incentive to switch back to the UP option, and should continue to choose it until the SP value reaches 12.00 (where they are predicted to be indifferent between the two options). This increase in switch frequency from level-1 play to levels 2 and 3 increases further for level-4 players (5% of players), which should in fact switch 9 times (and we truncated the distribution at k = 4). Importantly, these switching predictions can also hold when relaxing the assumption of Camerer *et al*.^20^ that subjects know the distribution of players. For instance, a level-k player that only believes others are randomly distributed over the possible k-1 types, leads to the same switching predictions as above for players levels 0 through 2, while level 3 players now switch 5 times instead of 3, and level 4 players switch 7 times instead of 9. In both cases, higher levels of reasoning can be associated with higher number of switches.

This characterization has at least two intriguing properties for our purposes: first, level-0 and level-4 players display similar rates of switching, thus similar levels of entropy, highlighting a theoretical difference, absent for SHs, between strategic entropy (e.g., level-4 players, but also levels 2 and 3) and non-strategic entropy (level-0 players), or between behavior that is random and behavior that may “look” random but is not. Second, the average switching rate of such a distribution of players is 3.9, which is very close to the average switching rates of 4 we observe. Relatedly, the distribution of switches predicted by the theory suggests that peaks should be observed at a threshold frequency of 1 (33% of level-1 players) and 3 (22% and 12% of level 2 and 3 players, respectively), which is also where we observe the two highest concentrations of players (respectively, 19% and 25%).

**Figure 5:**
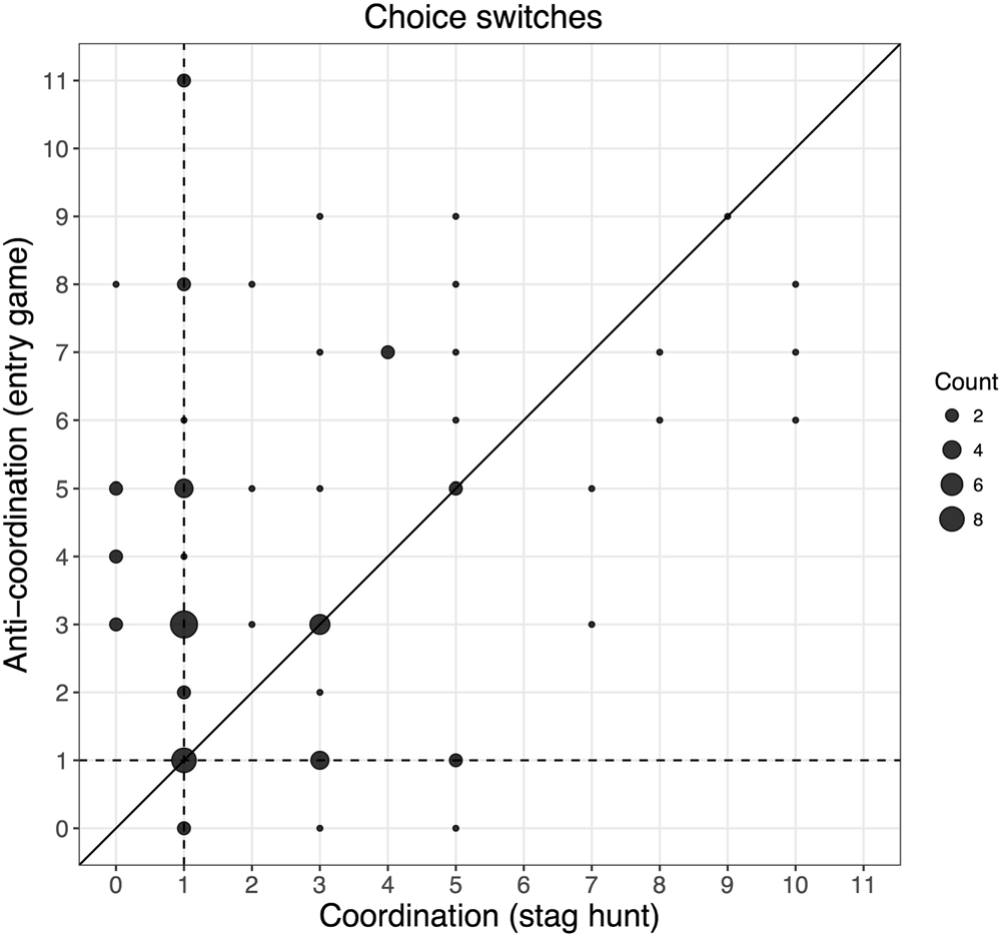
Choice switches in coordination and anti-coordination games (i.e., stag hunts and entry games, respectively). Circle areas indicate how many participants switched a given amount of times between choosing a safe option, as opposed to an uncertain option, as the value of the safe payoff was gradually increased. Observations along the dashed lines represent participants that adopted “perfect threshold strategies” (i.e., only one “choice switch”), in the stag hunts (horizontal dashed line) and in the entry games (vertical dashed line). We observe that perfect thresholds (i.e., one switch) are the modal frequency in stag hunts, used by 42% of participants, while this percentage drops to 19% in entry games (where the modal choice-switch frequency was 3, for 25% of participants). An equal number of choices switches across the two games would have resulted in observations lying on the main diagonal. The figure shows that the majority of participants increased switching frequency when passing from coordination to anti-coordination games, as most observations are above the diagonal.

We next asked whether the higher entropy levels of the entry game could be due to relatively few participants displaying a particularly high number of switches in this game. However, Fig. 5 suggests that this isn’t the case: the majority of subjects increased the number of choice switches when passing from coordination to anti-coordination games.

Finally, we find that the frequency of choice switches predicted expected earnings only for the entry game (r = 0.28, p < 0.05), while it decreased them in the two lotteries (r_s_ > 0.3, p_s_ < 0.01) (and non-significantly decreased them in the stag hunt).

## Discussion

In this study we provide an empirical answer to an old question: whether betting on others is perceived like betting on nature^1^. We conjectured that one potentially important difference between these two domains is that humans should think recursively (i.e., strategically) about other intentional/motivated agents but not about “blind nature”. We further hypothesized that, behaviorally, a sign of recursive reasoning should especially be detectable in an anti-coordination environment (i.e., in which choices can mutually offset one another, such as an entry game), rather than a more cooperative one involving coordination (in which choices can reinforce one another, such as a stag hunt). We investigated this by systematically comparing binary decisions between a safe option (i.e., a safe payoff that we progressively increased) and an uncertain option in four decision environments that differed only in the nature of the uncertainty and were identical in their monetary incentives: games against others (pure coordination vs. anti-coordination), and games against nature (risky vs. ambiguous lotteries). We find that i) in terms of certainty equivalents (i.e., the specific payoff-threshold that makes subjects indifferent between the two options), uncertainty is similarly high under anti-coordination and ambiguous lotteries, intermediate in the risky lotteries and lowest in stag hunts; ii) subjects switch between options more frequently, displaying higher entropy, under anti-coordination relative to any of the other tested environments; and that iii) subjects take more time to reach their decisions in anti-coordination games, relative to any other environment.

Our certainty equivalent results corroborate and extend previous findings suggesting that coordination and anti-coordination have an opposite impact on uncertainty. For instance, Camerer and Karjalainen^28^ observed that participants exhibit aversion to uncertainty in an anti-coordination game (i.e., a “miscoordination” game), while Fox and Weber^27^ observed affinity to uncertainty in a coordination game. Finally, Chark and Chew^26^ recently corroborated both of these findings by showing that subjects prefer to play a coordination game strategically, rather than non-strategically (i.e., when a random lottery makes the selection for the player), but that the opposite is true for anti-coordination environments. Our results depict an even more systematic picture: certainty equivalents are (much) higher in coordination games, intermediate in risky lotteries, and lowest in anti-coordination, with each condition differing significantly from the other.

We extend these previous findings in two ways. First, we show that, on average, subjects exhibit similarly high aversion to uncertainty in anti-coordination environments and ambiguous lotteries. We suggest that this could emerge because recursive reasoning changes choices in anti-coordination but not coordination games, decreasing subjects’ confidence about their ability to infer the choices of others. This is also in line with two studies that showed how removing ambiguity from the social context - by having participants coordinate with similar (vs. dissimilar) others^29^, or friends (vs. strangers)^30^, leads to affinity towards uncertainty in stag hunts, but to higher aversion towards uncertainty in entry games.

However, we believe that the most important insight afforded by our results, relative to those, is that certainty equivalents alone might provide an excessively restricted window into the decision processes underlying strategic uncertainty. Indeed, even though certainty equivalents are similar in competitive/anti-coordination games and ambiguous lotteries, we find that subjects make their decisions in a radically different way in these two decision domains: subjects “switched options”, much more frequently in the anti-coordination environment, relative to any of the other tested environments. The studies mentioned above^26–28^ did not measure entropy across the decision environments. The main novelty of our result is thus to dissociate between two measures: while some environments, such as ambiguity and anti-coordination, can elicit similar average certainty equivalents (i.e., “betting preferences”) they can elicit very different decision patterns in terms of entropy. We thus suggest that despite the similar levels of aversion to uncertainty, the effects of anti-coordinating with others should not be reduced to mere ambiguity-aversion (or source preference) in individual decision making^26^.

A parsimonious explanation of these results could be attributed to the fact that anti-coordination environments are simply more complex than coordination environments or lotteries. Indeed, complexity or task difficulty could account for the lower confidence (and thus higher entropy) of anti-coordination, as well as the increased decision times we observe in these environments. However, this interpretation does not seem to provide a clear answer as to what can make social decisions “complex”. In fact, at least superficially, stag hunts and entry games can be represented in a simple manner: both of our variants involved only two possible options, two players and four possible outcomes, yet anti-coordination resulted in very different behavioral patterns. Cognitive hierarchy theories might provide an explanation as to *why* “anti-coordinating” is more complex: recursive thinking only switches one’s choices in this domain and this can increase both the variance of one’s own response as well one’s estimates of the choices of others.

Moreover, our parametric modulation of the sure payoff could also suggest that the increased hierarchical reasoning that occurs in mismatching games is not circular, but follows a specific pattern that is predicted by cognitive hierarchy models^18,20^. In fact, extending our introductory examples, cognitive hierarchy models predict that, in both of our coordination games, (risk-neutral) level-1 players should always choose the UP option when the SP value is lower than half of the UP value (thus €7.50, since this is the expected value of a random lottery that pays €15.00 or 0 with probability 0.5). Level-2 players on the other hand believe they are interacting with a mixture of level-0 and level-1 players. Consequently, they expect their counterparts will most likely choose the UP for low SP values (i.e., SP <7.5) (since half level-0 players and all the level-1 players are doing this), but the UP at higher SP values (SP >7.5). As illustrated in the introduction, while this belief doesn’t change the behavior of level-2 players (or even higher level players) in stag hunts (relative to level-1 players), it does in entry games. Indeed, level-2 players in the entry game should choose the SP option for low SP values more frequently than level-1 players, as an evasion tactic to avoid the wave of lower level players who are expected to choose the UP in this SP range. By the same token, however, since level-2 players expect that relatively less lower-level players choose the UP at high SP values, they might choose the UP in such a SP range.

Our findings are in line with these cognitive hierarchy predictions. In fact, consistent with level-2 play, especially at low SP values (i.e., SP < 7.5), the probability of UP choices (see Fig. 3) was lowest in the anti-coordination game, relative to any of the other decision environments. Conversely, at high SP values, we observe a slight “kink” upwards in the entry game curve, relative to the corresponding lottery environments (thus relative to hypothetical level-1 play). This plausibly resulted from the fact that (in addition to the lower rate of UP choices at low SPs) some (i.e., level-2) players chose the UP even when the SP was relatively high (i.e., SP  > 7.50), resulting in an overall more slanted slope of the estimated logistic function fit to the entry game. Indeed, these players could be rather certain that, at such high SP ranges, most possible counterparts would choose the SP, thus reasoning one step forward and choosing the opposite. Intriguingly, previous research on cognitive hierarchy^31^ suggests that, on average, experimental participants exhibit levels of reasoning that approximate a Poisson distribution with lambda = 1.5, and the response curve we observe for the entry game seems in line with such a distribution of players. Moreover, we find that the aggregate switching frequency predicted by that lambda value resembles what we observe in the data. Future research could further address this by relating choices and switching patterns under anti-coordination to choices in other games, such as the p-beauty contest, in which levels of reasoning are easier to identify^18–20^.

At last, a possible alternative explanation to our results is that, in addition to hierarchical reasoning, there could be another potentially important difference between games against others and games against nature, namely, “social preferences”. Indeed, it can be formally shown that even simply adding an “altruism” parameter into the payoff function of a game - thus making agents (positively) interested in the payoff of their counterparts - can increase the attractiveness of the UP choices (relative to SP) in stag hunts, and of SP choices in entry games, which is at least consistent with our threshold findings. Our study was not intended to address this issue, however it is worth noting that while certain social preference models could account for our threshold findings, they would seem less at ease with the increased number of switches observed in EGs, as well as the increased decision times that occur in this domain. With notable exceptions^32,33^, most of these models are in fact - as the general term states - “social *preference*” models. Neither these nor “source *preference*” or ambiguity models^26^ explicitly feature the “social inferences”^34,35^ that underlie belief (and higher order belief) formation and that were the original objective of early coordination researchers^4^. For instance, Cooper & Dejong^4^ opened their seminal paper with the following: “A weakness of the Nash equilibrium concept for noncooperative games is that it may not generate a unique outcome. In this case it might be augmented by a hypothesis refining the *beliefs* of players about the strategies selected by their opponents” [cursive ours]. We speculate that a possible belief-refinement that could be consistent with our results is one that attributes a role to social-projection^36^, similarity^29,37–40^ or “expected mimicry”^41^, to initial belief formation in symmetric games (and similarity-sensitive games specifically^42^). In fact, if beliefs about others are anchored to one’s personally preferred outcomes, this could generate assurance in stag hunts (where agents arguably prefer the pareto-efficient option), while simultaneously increasing uncertainty in entry games.

In conclusion, our results suggest that, from a behavioral perspective, games against others and games against nature are not treated similarly: in anti-coordination environments, humans exhibit exceptionally high degrees of “choice switching” (or entropy), relative to environments that require them to match/coordinate their choices, or to bet on (risky or ambiguous) lotteries with identical monetary incentives. We suggest that this behavioral pattern emerges from hierarchical reasoning about others in interdependent decision problems, something that does not apply to games against nature (and does not alter choices in stag hunts). Indeed, it would be interesting to individuate a one-shot game with nature capable of eliciting similar entropy levels as the ones observed here in the entry games. Our findings also suggest that different skills or strategies may underlie cooperative and competitive coordination, something which could also be in line with evolutionary and neuroscientific evidence^43–46^. In cooperative environments requiring subjects to tacitly coordinate their choices, recursive reasoning about others does not necessarily affect one’s choices, while it can lead to potentially strategic entropy in competitive situations requiring subjects to anti-coordinate their choices.

## Methods

### Participants

In 4 experimental sessions, 75 students (35 females, mean age = 23.4, SD = 3.7) took part in the tasks of interest. The experiment was conducted at the CEEL lab (Computable and Experimental Economics Laboratory) of the University of Trento (Italy) and participants were recruited via email through the lab’s database. All participants provided informed consent for the treatment and publication of their anonymized data. All assessments were approved by the Research Ethics Committee (agreement number 2008-008, “The neural bases of individual and strategic uncertainty”) of the University of Trento, Italy. All experiments were performed in accordance with relevant guidelines and regulations. The datasets generated during and/or analysed during the current study are available from the corresponding author on reasonable request.

### Procedures

As soon as they entered the lab, participants were designated to a computer cubicle (by extracting a bingo chip from a bag). Here, they found the paper instructions to the games, which were also read out loud by the experimenters. The instructions introduced the four decision environments of interest: two-player SHs and EGs, as well as risky and ambiguous lotteries, each of which was described in a neutral manner. Participants were explained that, in all environments, they would be required to choose between a certain payoff (“SP”), worth a given euro amount, and a potentially higher paying but uncertain payoff (“UP”), which was always worth either €15.00 or 0. In all environments, if participants chose the SP, they would earn that euro amount no matter what, while the choosing the UP had different consequences in different environments. SHs were indicated by the label “if at least 2” (see Fig. 1): if participants chose the UP in this game, they would earn the maximum earning (i.e., €15.00) *only if* a randomly selected counterpart had also chosen the UP, and 0 otherwise. Conversely, in EGs, labeled as “if at most 1”, if participants chose the UP, they would earn the maximum earning *only if* their counterpart had “not” chosen the UP; that is, if both participants chose the UP, both earned 0.

Risky lotteries were indicated by a figure representing an “urn” containing one red and one blue ball (see Fig. 1). Outside of the urn, a third ball indicated the “winning color” for the given trial (red or blue, the color was randomized). Participants were informed that if they chose the UP in this environment, their outcome would depend on a single blind extraction (performed by a randomly designated participant at the end of the experiment). Specifically, they would earn the maximum if the color of the extracted ball was the winning color, and 0 otherwise. This urn was physically implemented in front of the participants. Specifically, we ostensibly placed one blue ball and one red ball in an initially empty, opaque, cardboard box (with a hole on the top for later extraction), and then left it in sight until the end of the session, when the single extraction would take place. We also prepared a second, ambiguous urn (called “Urn 2”), in which we placed two red balls and two blue balls. Then we extracted two of the balls but did *not* show participants what color they were. Consequently, participants knew exactly the success probability of the risky lottery (p = 0.5), which we used as a baseline probability, but not of the ambiguous lottery (which could be either p = 1, p = 0, or p = 0.5). At last, participants were informed that they would make a number of decisions in each of these environments, that they would be re-matched with a random participant in each trial, and that they would not receive feedback on any of their decisions, until the end of the experiment, when a single trial would randomly be extracted and be paid (in private) according to actual choices.

After reading the instructions, participants underwent a thorough comprehension questionnaire that could only be concluded by correctly answering each of the questions. We also followed participants’ responses from the experimenter’s booth and assisted those that made any errors during this comprehension phase. At the end of this instruction phase we told participants that they had now all answered correctly to the questions and thus that the games were clear to all. To further ensure participants that no deception would take place, one participant was randomly selected (again via bingo chip) to be a monitor. The monitor followed participants’ anonymized responses from the experimenter’s booth in real time and paid them according to their actual choices, in cash, at the end of the session. No deception took place throughout the experiment.

### Game parameters

As mentioned above, in all environments and in all trials, the UP was always worth either €15.00 or 0. On the other hand, the monetary value of the SP varied in each trial, though, in all environments it was randomly extracted (without reinsertion) from the same set of possible values SP values: $SP\,\in \{1,\,2,\,3,\,4,\,5,\,6,\,6.5,\,7,\,7.5,\,8,\,8.5,\,9,\,9.5,\,10,\,11,\,12,\,13,\,14,\,15\}$. SP values thus covered a range between €1.00 and €15.00, in steps of 1 (with additional €0.5 values between €6.00 and €10.00). Each of these 19 SP values (and consequently each possible SP-UP pairing) was shown only once, for each participant, and for each decision environment. It follows that, in total, participants made 19 (SPs) * 4 (decision environments) = 76 decisions. The order of these 76 unique decisions was fully randomized.

### Measures

To investigate whether participants made choices differently in each of these domains, we compared various measures between each of the environments.

**Figure 6:**
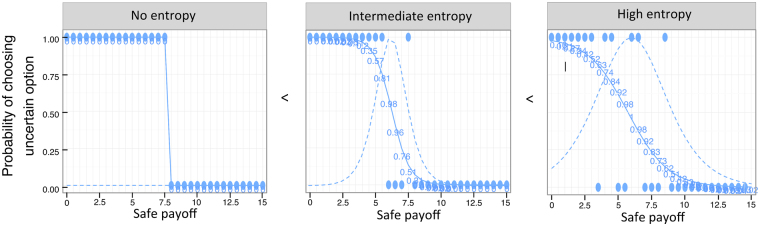
Illustration of entropy. Circles represent choices (1 = uncertain option, 0 = safe payoff) by three different participants. Solid lines are logistic fits to those choices, estimating the probability of choosing a potentially high paying but uncertain option, relative to a lower paying but safe alternative. Dashed lines represent the entropy related to the same logistic fits. The participants in the left most panel uses a “perfect threshold” strategy (thus, he/she exhibits no entropy). The participants in the middle and right panels have similar thresholds to the one on the left, yet they exhibit progressively higher levels of entropy.

As a first proxy for uncertainty, we investigated whether the probability of choosing the UP (as opposed to the SP) was affected by increasing SP values and their interaction with the environment (see below for statistical details). Then, following Heinemann and colleagues^11^, and as a more formal proxy for uncertainty, we computed certainty equivalents. To do this, we looped through each participants’ choices in each of the four environments, using a logistic function (equation 1) to estimate that participant’s probability of choosing the UP (“*P*(*UP*)”), given a particular SP value (see solid blue lines in Fig. 6, which illustrates the result of this procedure for three participants):

1 $$P(UP)=\frac{1}{1+{e}^{-({\beta }_{0}+{\beta }_{1}SP)}},$$

This yielded one intercept estimate (β_0_ indicating the probability of choosing the UP when the SP was 0) and one slope estimate (β_1,_ indicating how much the same probability was affected by a single unit change in the SP value), for each participant/environment. In this context, certainty equivalents (“CE”) could thus simply be computed as

2 $${\rm{CE}}\,=\,-{\beta }_{0}/{\beta }_{1},$$

and they indicated the estimated SP value that made subjects indifferent between choosing the SP and the UP. This resulted in N (participants) * 4 (environments) certainty equivalents. In 34 cases out of 300, we observed certainty equivalents that were out of range. This could occur for various reasons. For instance, if a participant always/never chose the UP in a given environment this resulted in certainty equivalents tending to +/− infinity. We handled these cases in two ways. In a first approach, we manually corrected such out of range certainty equivalents, by setting the ones that were lower than 0 to 0 and those that were higher than 15.00 to 15.00. In a second, we excluded these certainty equivalents altogether (thus labeling them as missing values). Since our final results were completely unaffected by this distinction, we disregard this matter and report results from the first of these two approaches.

To investigate whether subjects switched back and forth between the SP and UP option, as SPs increased, we computed two related measures. First, we computed entropy measures by applying the following binary entropy function (equation 3, below) to the vector of probabilities of choosing the UP option (obtained from the logistic fits above):

3 $$Entropy=-p(UP)lo{g}_{2}p(UP)-(1-p(UP))lo{g}_{2}(1-p(UP))$$

This function is maximal (i.e., 1) when subjects’ estimated p(UP) was equal to 0.5, and it decreases both for increasing and decreasing probabilities. Since the entropy function gives infinite values for p(UP) of 0 or 1, these were manually set to 0. As can be seen in Fig. 6 (Fig. 6), participants can have roughly the same certainty equivalents while still exhibiting a noticeable heterogeneity in entropy. Having obtained an entropy measure for each data point, we aggregated them over the SP values, for each participant/environment, thus again obtaining N*4 measures of entropy.

In addition to this, we counted “choice switches” that we expected to be raised by recursive reasoning in games with strategic substitutability. Specifically, we ordered trials by SP values in ascending order (for each participant/environment). Normally, participants choose the UP for low SP values and shift to choosing the SP option at some higher value of the SP^11^. In a perfect threshold strategy, this should occur only once (e.g., for perfect/deterministic threshold strategies), though often subjects exhibit imperfect threshold strategies, or non-threshold strategies, switching back and forth between SP and UP, as SP values increase^47^. We thus proceeded to measure this by simply counting the number of switches exhibited by each participant in each environment (as opposed to analyzing the continuous entropy measure described above). Notably, these counts correlated strongly with the entropy estimates (with a correlation coefficient of R = 0.94, p < 0.001), and all of the results reported above for one measure also held for the other.

To obtain a proxy for performance in our decision tasks, we investigated how coordination an anti-coordination affected expected earnings. To compute the latter, we did the following. In all the environments, if a participant chose the SP, the corresponding SP value was attributed to him or her, since this depended on nothing else. On the other hand, if one chose the UP, his or her expected payoff depended on two aspects: first, what game was being played (e.g., coordination or anti-coordination) and second, what others chose. Thus, in the stag hunt, since one had equal chances of being matched to any counterpart, we computed the expected payoff as

4 $$E{V}_{i(UPx)}^{Stag}=15.00\ast \frac{{\sum }_{-i}UP(x)}{n-1},$$

where the left side of the equation is the expected value of a UP choice in a stag hunt for participant “i” on trial “x”; while the right side of the equation is simply the proportion of (non-i) participants who also chose the UP on the same trial (x) and “n” is the total number of participants (minus one to exclude participant “i”). In short, if one chose the UP in the stag hunt, his or her expected payoff is proportional to the number of others who also chose the UP on the same trial. On the other hand, the expected value of choosing the UP in the entry game was simply computed as

5 $$E{V}_{i(UPx)}^{Entry}=15.00\ast (1-\frac{{\sum }_{-i}UP(x)}{n-1}),$$

since, in this case, one’s chances of earning the high €15.00 prize was inversely proportional to the number of others who also chose the UP on the target trial. Having obtained these measures of expected earnings, we aggregated them over the SP values, so as to again obtain one measure for each participant and environment.

Finally, as an additional proxy for the hypothesized (recursive) reasoning of entry games, relative to the other environments, we investigated the impact of the tested decision environments on response times (of which we took the log, to better align with normality assumptions). Due to a programming error, the response times of two sessions were not recorded. However, Rubinstein^22^ suggests that typical response time experiments involve roughly 20 participants, and 100–200 trials per participant. We have fewer trials (76) but more participants (39 participants.), suggesting that the decision time results are reliable.

### Statistical analyses

Following Heinemann and colleagues^11^, we used logistic hierarchical models^48^ to cluster choices by participant, which were treated as random effects, and to estimate fixed effects (i.e., the dichotomous choice of UP or SP) on the basis of the following predictors: the decision environment (4 levels: risky lottery, ambiguous lottery, stag hunt and entry game), the sure payoff term, and the interaction between this and the decision environment. Further comparisons between environments were carried out with Wilcoxon signed-rank tests. All analysis were carried out in R^49^.
